# Competing risk nomogram predicting cancer‐specific mortality for endometrial cancer patients treated with hysterectomy

**DOI:** 10.1002/cam4.3887

**Published:** 2021-05-01

**Authors:** Guilan Xie, Cuifang Qi, Wenfang Yang, Ruiqi Wang, Liren Yang, Li Shang, Liyan Huang, Mei Chun Chung

**Affiliations:** ^1^ Department of Obstetrics and Gynecology, Maternal and Child Health Center The First Affiliated Hospital of Xi'an Jiaotong University Xi'an China; ^2^ School of Public Health Xi'an Jiaotong University Health Science Center Xi'an China; ^3^ Department of Public Health and Community Medicine Tufts University School of Medicine Boston Massachusetts USA

**Keywords:** competing risk, decision curve analysis, endometrial cancer, nomogram, prognosis

## Abstract

**Background:**

The incidence of endometrial cancer has tended to increase in recent years. However, competing risk nomogram combining comprehensive factors for endometrial cancer patients treated with hysterectomy is still scarce. Therefore, we aimed to build a competing risk nomogram predicting cancer‐specific mortality for endometrial cancer patients treated with hysterectomy.

**Methods:**

Patients diagnosed with endometrial cancer between 2010 and 2012 were abstracted from the Surveillance, Epidemiology, and End Results (SEER) database. Competing risk model was performed to select prognostic variables to build the competing risk nomogram to predict the cumulative 3‐ and 5‐year incidences of endometrial cancer‐specific mortality. Harrell's C‐index, receiver operating characteristic (ROC) curve, and calibration plot were used in the internal validation. And decision curve analysis was applied to evaluate clinical utility.

**Results:**

A total of 10,447 patients were selected for analysis. The competing risk nomogram identified eight prognostic variables, including age at diagnosis, race, marital status at diagnosis, grade, histology, tumor size, FIGO stage, and number of regional nodes positive. The C‐index of the competing risk nomogram was 0.857 (95% confidence interval [CI]: 0.854–0.859), and the calibration plots were adequately fitted. When the threshold probabilities were between 1% and 57% for 3‐year prediction and between 2% and 67% for 5‐year prediction, the competing risk nomogram was of good clinical utility.

**Conclusions:**

A competing risk nomogram for endometrial cancer patients treated with hysterectomy was successfully built and internally validated. It was an accurately predicted and clinical useful tool, which could play an important role in consulting and health care management of endometrial cancer patients.

## Graphical abstract

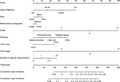

## 1 INTRODUCTION

Endometrial cancer is one of the three common malignancies about female reproductive system, and its incidence has tended to increase in recent years.^1^ Its prognosis is relatively good, because most patients are diagnosed at early stages and treated with hysterectomy therapy. It is estimated that the 5‐year relative survival rate of endometrial cancer is 83.4%, but its survival rate has no significant improvement since the mid‐1970 s.^2^ Some studies proved that, in addition to International Federation of Gynecology and Obstetrics (FIGO) stage, demographic and tumor‐related information also had effects on the prognosis of endometrial cancer.^3^, ^4^, ^5^

Generally, Kaplan–Meier method and Cox regression model are widely used in studies with survival analysis, which classify outcomes into two categories, dead and censored. However, if patients die for other causes, the competing events would impede the occurrence of interest event and change the probability of interest event.^6^ The Kaplan–Meier method fails to be used in survival data with multiple outcomes and the Cox regression model may lead to deviation of the predicted probability of interest event for ignoring the compering risks by deeming competing events as censorship. Therefore, traditional methods of survival analysis are not satisfactory enough when there are competing risks. Cumulative incidence function and competing risk model can handle survival data that have multiple endpoint events with competing relationships. Because of taking competing risks into account, the model can explore the relationships between factors and prognosis more accurately than traditional methods of survival analysis,^7^ and it has gradually been used in researches with survival data.^8^, ^9^, ^10^

Nomogram is a calculation tool based on the complex formula of prediction model, which can quantify the impacts of various factors. Currently, it has been used to visualize the prediction models of cancers. Several studies have also used nomogram to predict the prognosis of endometrial cancer.^11^, ^12^, ^13^ However, most of them used Kaplan–Meier method to plot survival curves and Cox regression model to build prediction models, and the application of cumulative incidence function and competing risk model was still rare. And most prediction models lacked the test of clinical benefit, which was the key to determine whether the prediction model was of actual use value. Besides, multiple major journals have published recommendations of decision curve analysis, which could measure the clinical usefulness of prediction models.^14^, ^15^

Herein, we aimed to build a competing risk nomogram incorporating demographic and tumor‐related information to predict cancer‐specific mortality for endometrial cancer patients treated with hysterectomy.

## 2 MATETIALS AND METHODS

### 2.1 Study population

In this retrospective study, patients (*n* = 10,447) diagnosed with endometrial cancer between 2010 and 2012 were abstracted from the population‐based Surveillance, Epidemiology, and End Results (SEER) database. The SEER database is publicly available and currently has 18 cancer registries collecting cancer diagnosis, treatment, and survival data for approximately 28% of the US population.^16^

Patients who were older than or equal to 18 years old, with histological clear diagnosis of primary endometrial cancer, treated with hysterectomy, and whose data were not obtained from autopsy or death certificate were included. Patients with unknown information related to demographic and endometrial cancer or incomplete follow‐up information were excluded. Besides, those with survival time less than 1 month were also excluded, for they tended to have serious comorbidities. The flowchart of data selection was provided in Figure S1.

### 2.2 Predictive variables

The variables collected in this study contained age at diagnosis, race (white, black, and other), marital status at diagnosis (single, married, and other), grade (I [well differentiated], II [moderately differentiated], III [poorly differentiated], and IV [undifferentiated or anaplastic]), histology (adenocarcinoma, carcinosarcoma, clear cell, papillary serous, and other), tumor size, FIGO stage (I, II, III, and IV), and number of regional nodes positive. Some continuous variables were further categorized, containing age at diagnosis (<50, 50–59, 60–69, ≥70 [years old]), tumor size (≤3 and >3 cm), and number of regional nodes positive (0, 1, 2–5, and >5).

### 2.3 Outcomes

The final follow‐up status was divided into endometrial cancer‐specific mortality, other causes‐specific mortality and censored. Death due to primary endometrial cancer was deemed as endometrial cancer‐specific mortality. Death owing to non‐cancer causes and other cancers were deemed as other causes‐specific mortality. Other cases were defined as censored data. The period from being diagnosed to follow‐up endpoint was survival time.

### 2.4 Statistical analysis

Chi‐square test was used to describe the distributional differences. Among these survival outcomes, endometrial cancer‐specific mortality was the interest event, while other causes‐specific mortality was the competing event. Cumulative incidence of function was applied to calculate the cumulative incidence of events. Competing risk model was performed in the univariate and multivariate competing risk analysis to find the associations between predicted variables and endometrial cancer‐specific mortality. And subdistribution hazard ration (SHR) was estimated to explore the relationships between each subgroup and interest event. Variables, which were of clinical significance and with *p* value less than 0.05 in the univariate competing risk analysis, were involved in the multivariate competing risk analysis. And stepwise regression was used in multivariate competing risk analysis in order to find out the significant variables without serious multicollinearity of the model. The eventually selected variables were used to build the competing risk nomogram.

To measure the performance of the competing risk nomogram, bootstrap 1000 resampling and 10‐fold cross‐validation were performed in the internal validation. Discrimination of the competing risk nomogram was signified by Harrell's concordance index (C‐index), which indicated whether the competing risk nomogram could correctly distinguish interest event from censored and competing events. C‐index ranged from 0 to 1. When C‐index was 0.5, it illustrated that there was a random relationship between predicted and real probability. And the nomogram was considered of great discrimination when the C‐index was close to 1. In addition, the area under the receiver operating characteristic (ROC) curve (AUC) was adopted to quantify the discrimination performance of the competing risk nomogram in 3‐ and 5‐year prediction.

Calibration plot was used to measure the consistency between predicted and observed probabilities of endometrial cancer‐specific mortality. In the calibration plot, we sorted the predicted probabilities from small to large and divided them into 10 groups according to the deciles. The average of predicted probabilities for each group was displayed on the *X* coordinate, while the proportion of actual events was displayed on the *Y* coordinate. The 45° diagonal line represented that the predicted probabilities were equal to the actual probabilities. The closer the calibration plot was to the ideal line, the better the calibration of the prediction model.

Eventually, decision curve analysis was performed to evaluate clinical utility of the competing risk nomogram, which could put sight into the net benefit of competing risk nomogram. In decision curve, there were two situations; all patients were treated with the competing nomogram and none. The horizontal line indicated the situation when none patients was treated and the slanted line with a negative slope indicated the situation when all patients were treated. *X* coordinate represented threshold probability and *Y* coordinate represented net benefit, which was gained by subtracting harms from benefits.

All analyses were conducted with the “mstate,” “rms,” “cmprsk,” “riskRegression,” “pec,” “timeROC,” and “rmda” packages of R 3.6.1 (https://www.r‐project.org/). Two‐tailed *p* value <0.05 indicated significance.

## 3 RESULTS

### 3.1 Baseline characteristics

**FIGURE 1:**
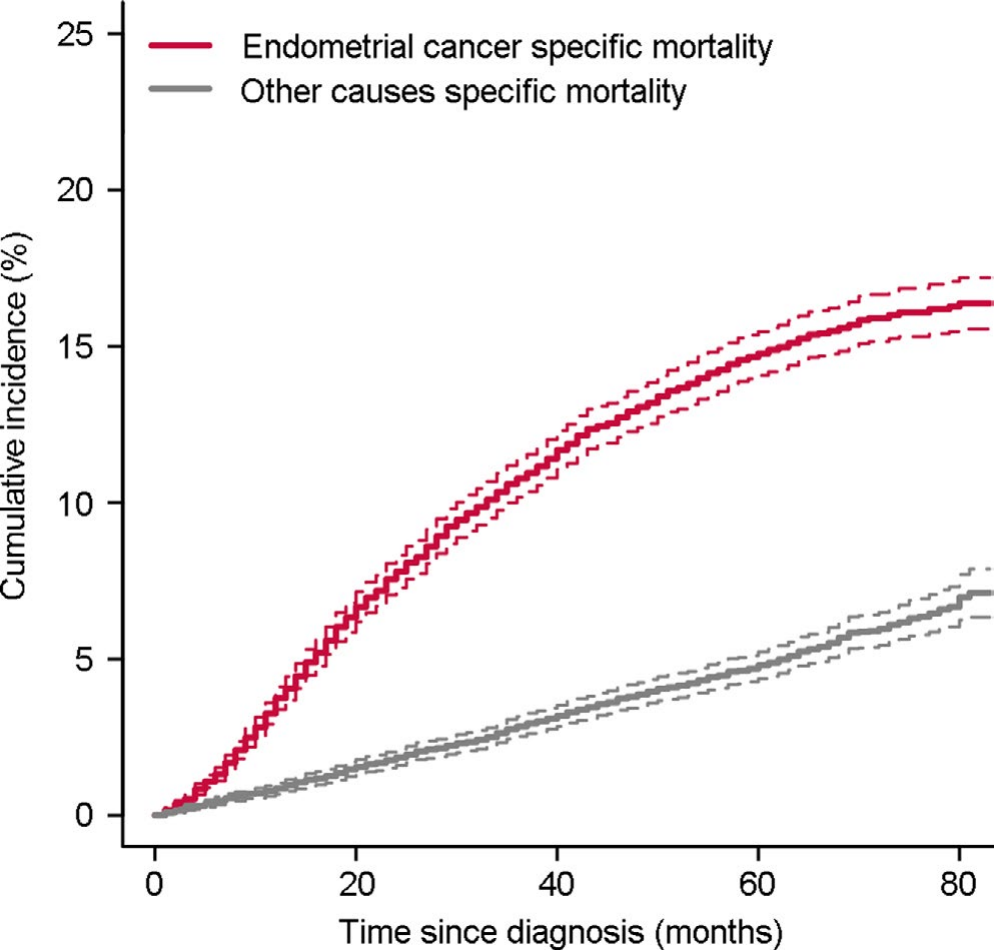
Cumulative incidence curve of endometrial cancer specific mortality and other causes specific mortality. Dashed lines were 95% confidence interval

A total of 10,447 patients were included for analysis. Among them, 8371 (80.13%) patients were censored, 1535 (14.69%) patients were dead of endometrial cancer and 541 (5.18%) patients were dead of other causes. Baseline characteristics of patients with different outcomes was shown in Table 1. The differences between different outcomes were statistically significant in all variables. The median follow‐up time were 74.16 (95% CI: 73.74–74.58) months. The cumulative incidence of death increased over time and the cumulative incidence of endometrial cancer‐specific mortality was higher than other cause‐specific mortality. The cumulative 3‐year incidence of endometrial cancer‐specific mortality was 10.764% (95% CI: 10.762%–10.766%), and other cause‐specific mortality was 2.843% (95% CI: 2.843%–2.844%). The cumulative 5‐year incidence of endometrial cancer‐specific mortality was 14.767% (95% CI: 14.765%–14.770%), and other cause‐specific mortality was 4.795% (95% CI: 4.794%–4.796%). The cumulative incidence curve was showed in Figure 1.

**TABLE 1:** Baseline characteristics of endometrial cancer patients treated with hysterectomy

Variables	Total number (*n* = 10447)	Censored (*n* = 8371)	Endometrial cancer specific mortality (*n* = 1535)	Other causes specific mortality (*n* = 541)	*χ* ^2^	*p* value
Age at diagnosis (years old), *n* (%)					530.89	<0.001
<50	1062 (10.16)	963 (11.50)	79 (5.15)	20 (3.70)		
50–59	3071 (29.40)	2657 (31.74)	331 (21.56)	83 (15.34)		
60–69	3811 (36.48)	3083 (36.83)	588 (38.31)	140 (25.88)		
≥70	2503 (23.96)	1668 (19.93)	537 (34.98)	298 (55.08)		
Race, *n* (%)					163.14	<0.001
White	8423 (80.63)	6843 (81.75)	1133 (73.81)	447 (82.62)		
Black	949 (9.08)	623 (7.44)	261 (17.00)	65 (12.02)		
Other	1075 (10.29)	905 (10.81)	141 (9.19)	29 (5.36)		
Marital status at diagnosis, *n* (%)					153.84	<0.001
Single	2050 (19.62)	1641 (19.60)	308 (20.06)	101 (18.67)		
Married	5664 (54.22)	4746 (56.70)	699 (45.54)	219 (40.48)		
Other	2733 (26.16)	1984 (23.70)	528 (34.40)	221 (40.85)		
Grade, *n* (%)					1373.75	<0.001
I	3551 (33.99)	3289 (39.29)	124 (8.08)	138 (25.51)		
II	3415 (32.69)	2928 (34.98)	308 (20.07)	179 (33.09)		
III	2609 (24.97)	1687 (20.15)	756 (49.25)	166 (30.68)		
IV	872 (8.35)	467 (5.58)	347 (22.60)	58 (10.72)		
Histology, *n* (%)					905.70	<0.001
Adenocarcinoma	9310 (89.12)	7804 (93.22)	1042 (67.88)	464 (85.77)		
Carcinosarcoma	352 (3.37)	153 (1.83)	178 (11.60)	21 (3.88)		
Clear cell	131 (1.25)	82 (0.98)	39 (2.54)	10 (1.85)		
Papillary serous	292 (2.80)	138 (1.65)	128 (8.34)	26 (4.80)		
Other	362 (3.46)	194 (2.32)	148 (9.64)	20 (3.70)		
Tumor size, *n* (%)					270.85	<0.001
≤3 cm	3917 (37.49)	3458 (41.31)	310 (20.20)	149 (27.54)		
>3 cm	6530 (62.51)	4913 (58.69)	1225 (79.80)	392 (72.46)		
FIGO stage, *n* (%)					1726.78	<0.001
I	7613 (72.87)	6693 (79.95)	548 (35.70)	372 (68.76)		
II	639 (6.12)	480 (5.74)	116 (7.56)	43 (7.95)		
III	1792 (17.15)	1078 (12.88)	609 (39.67)	105 (19.41)		
IV	403 (3.86)	120 (1.43)	262 (17.07)	21 (3.88)		
Number of regional nodes positive, *n* (%)					1204.89	<0.001
0	8906 (85.25)	7560 (90.31)	884 (57.59)	462 (85.40)		
1	579 (5.54)	355 (4.24)	198 (12.90)	26 (4.81)		
2–5	695 (6.65)	367 (4.39)	294 (19.15)	34 (6.28)		
>5	267 (2.56)	89 (1.06)	159 (10.36)	19 (3.51)		

### 3.2 Prognostic variables

The results of competing risk analysis were outlined in Table 2. All variables were statistically significant associated with endometrial cancer‐specific mortality both in the univariate and multivariate competing risk analysis. The FIGO stage was the predictive variable with the highest subdistribution hazard ratio. In the univariate competing risk analyses, compared with FIGO Stage I, all the Stage II (SHR: 2.69, 95% CI: 2.21–3.28, *p *< 0.001), Stage III (SHR: 5.60, 95% CI: 4.99–6.29, *p *< 0.001), and Stage IV (SHR: 14.97, 95% CI: 12.86–17.44, *p *< 0.001) had higher risks of endometrial cancer‐specific mortality. In the multivariate competing risk analyses, compared with FIGO Stage I, all the Stage II (SHR: 2.01, 95% CI: 1.64–2.47, *p *< 0.001), Stage III (SHR: 2.62, 95% CI: 2.18–3.13, *p *< 0.001), and Stage IV (SHR: 5.88, 95% CI: 4.81–7.18, *p* < 0.001) still had higher risks of endometrial cancer‐specific mortality. Besides, grade was also a critical predictive variable. In the univariate competing risk analyses, compared with Grade I, Grade II (SHR: 2.64, 95% CI: 2.15–3.25, *p* < 0.001), Grade III (SHR: 9.63, 95% CI: 7.97–11.64, *p* < 0.001), and Grade IV (SHR: 14.71, 95% CI: 11.98–18.07, *p* < 0.001) had higher risks of endometrial cancer‐specific mortality. In the multivariate competing risk analyses, compared with Grade I, Grade II (SHR: 1.98, 95% CI: 1.60–2.44, *p* < 0.001), Grade III (SHR: 4.27, 95% CI: 3.48–5.24, *p* < 0.001), and Grade IV (SHR: 5.08, 95% CI: 4.03–6.39, *p *< 0.001) still had higher risks of endometrial cancer‐specific mortality.

**TABLE 2:** Univariate and multivariate competing risk analysis for endometrial cancer specific mortality

Variable	Univariate competing risk analysis		Multivariate competing risk analysis	
	SHR (95% CI)	*p* value	SHR (95% CI)	*p* value
Age at diagnosis (years old)				
<50	Reference		Reference	
50–59	1.45 (1.14,1.86)	0.003	1.44 (1.12,1.85)	0.005
60–69	2.09 (1.66,2.65)	<0.001	1.83 (1.43,2.34)	<0.001
≥70	3.02 (2.39,3.83)	<0.001	2.40 (1.87,3.09)	<0.001
Race				
White	Reference		Reference	
Black	2.24 (1.96,2.55)	<0.001	1.31 (1.13,1.52)	<0.001
Other	1.00 (0.84,1.19)	0.990	1.00 (0.84,1.20)	0.970
Marital status at diagnosis				
Single	Reference		Reference	
Married	0.81 (0.71,0.92)	0.002	0.85 (0.73,0.98)	0.022
Other	1.31 (1.14,1.51)	<0.001	1.05 (0.90,1.22)	0.540
Grade				
I	Reference		Reference	
II	2.64 (2.15,3.25)	<0.001	1.98 (1.60,2.44)	<0.001
III	9.63 (7.97,11.64)	<0.001	4.27 (3.48,5.24)	<0.001
IV	14.71 (11.98,18.07)	<0.001	5.08 (4.03,6.39)	<0.001
Histology				
Adenocarcinoma	Reference		Reference	
Carcinosarcoma	6.41 (5.44,7.57)	<0.001	2.23 (1.85,2.68)	<0.001
Clear cell	3.05 (2.20,4.24)	<0.001	0.98 (0.70,1.38)	0.920
Papillary serous	4.57 (3.84,5.45)	<0.001	1.50 (1.22,1.84)	<0.001
Other	4.74 (3.96,5.68)	<0.001	2.18 (1.78,2.67)	<0.001
Tumor size				
≤3 cm	Reference		Reference	
>3 cm	2.57 (2.27,2.91)	<0.001	1.43 (1.26,1.63)	<0.001
FIGO stage				
I	Reference		Reference	
II	2.69 (2.21,3.28)	<0.001	2.01 (1.64,2.47)	<0.001
III	5.60 (4.99,6.29)	<0.001	2.62 (2.18,3.13)	<0.001
IV	14.97 (12.86,17.44)	<0.001	5.88 (4.81,7.18)	<0.001
Number of regional nodes positive				
0	Reference		Reference	
1	3.99 (3.42,4.65)	<0.001	1.22 (1.00,1.50)	0.052
2–5	5.32 (4.66,6.08)	<0.001	1.44 (1.19,1.75)	<0.001
>5	9.19 (7.74,10.92)	<0.001	1.87 (1.49,2.34)	<0.001

### 3.3 Competing risk nomogram

**FIGURE 2:**
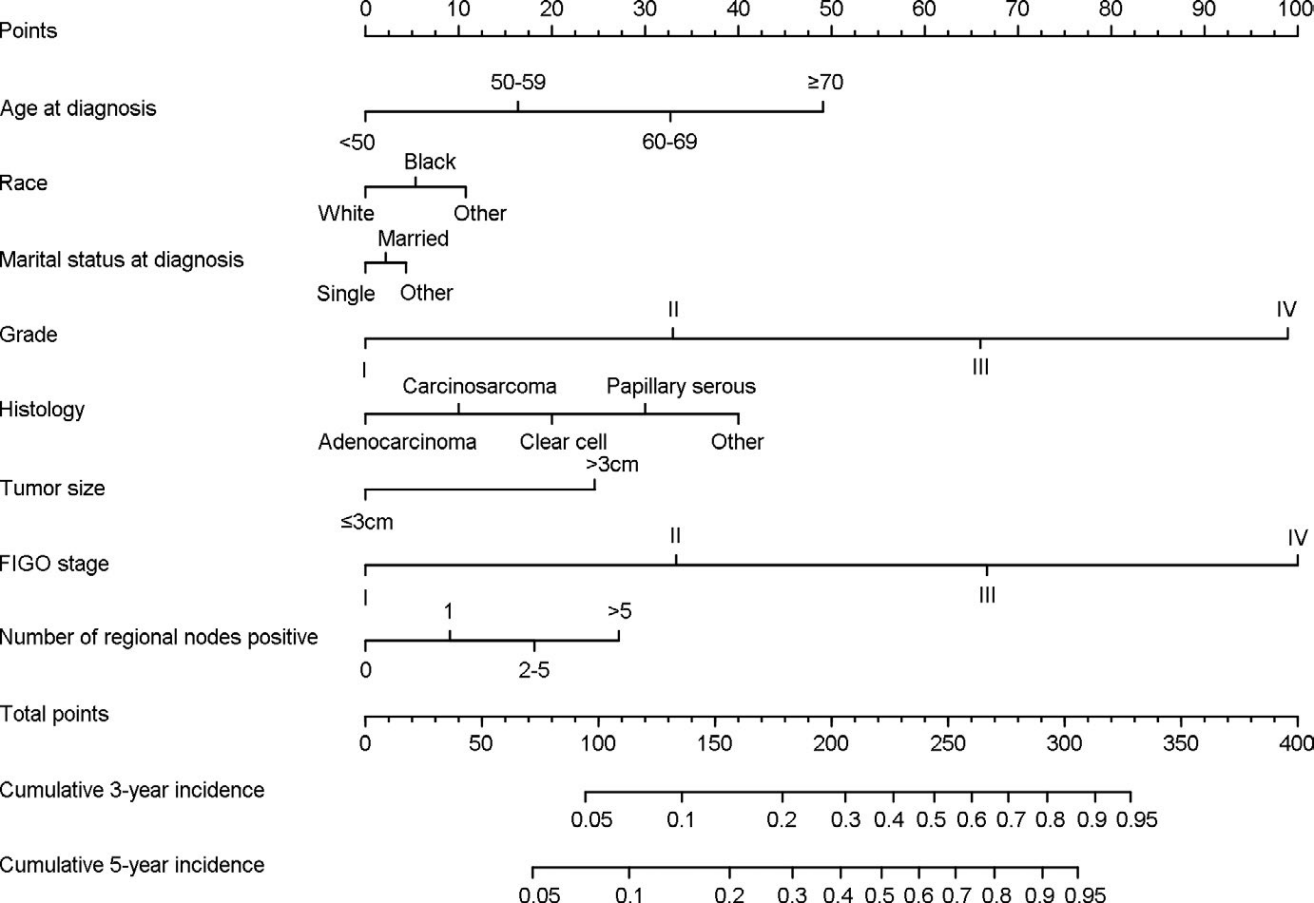
Competing risk nomogram of endometrial cancer specific mortality for endometrial cancer patients treated with hysterectomy

Finally, the competing risk nomogram was composed by eight prognostic variables, including age at diagnosis, race, marital status at diagnosis, grade, histology, tumor size, FIGO stage, and number of regional nodes positive (Figure 2). In the competing risk nomogram, FIGO stage and grade were the two most important predictive variables, which were significantly associated with endometrial cancer‐specific mortality. Besides, age at diagnosis, histology, and number of regional nodes positive followed them in turn. Marital status at diagnosis had minimal contribution to the model. The competing nomogram was used by calculating total points by summing up points of each variable in the top points scale. Cumulative 3‐ and 5‐year incidences of endometrial cancer‐specific mortality corresponding to total points scale were in the bottom of the competing risk nomogram.

### 3.4 Internal validation

**FIGURE 3:**
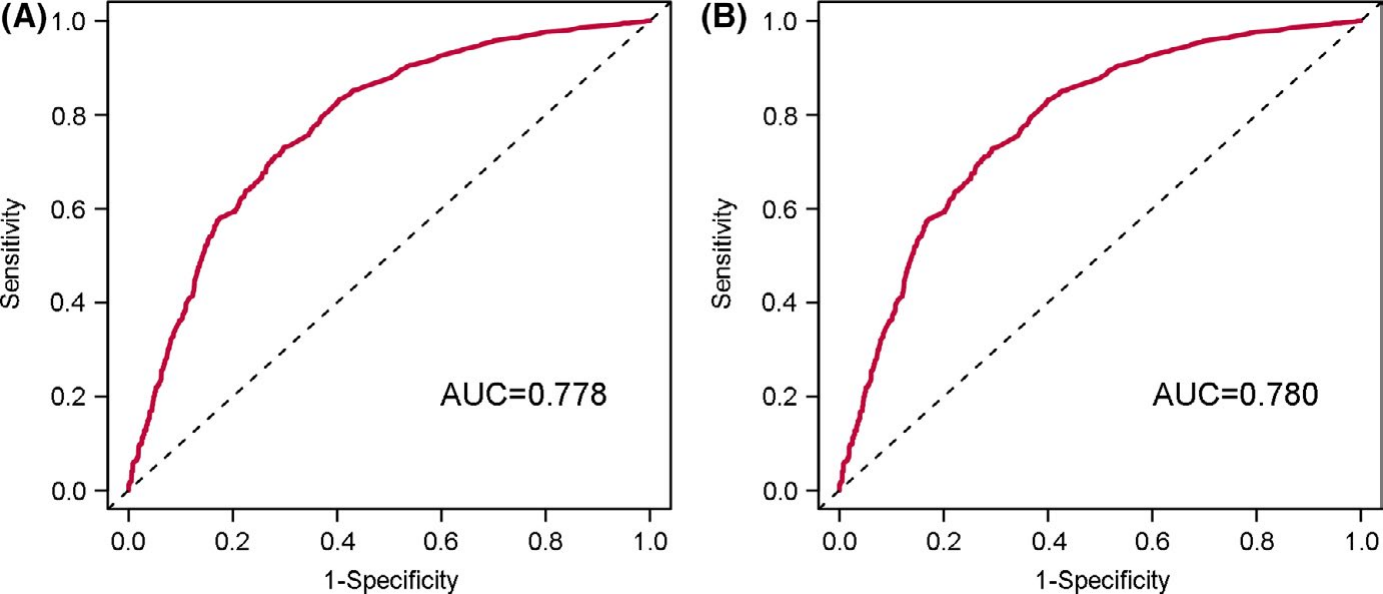
ROC curves for the competing risk nomogram. The ROC curves for the competing risk nomogram about (A) 3‐ and (B) 5‐year incidence of endometrial cancer specific mortality for endometrial cancer patients treated with hysterectomy

**FIGURE 4:**
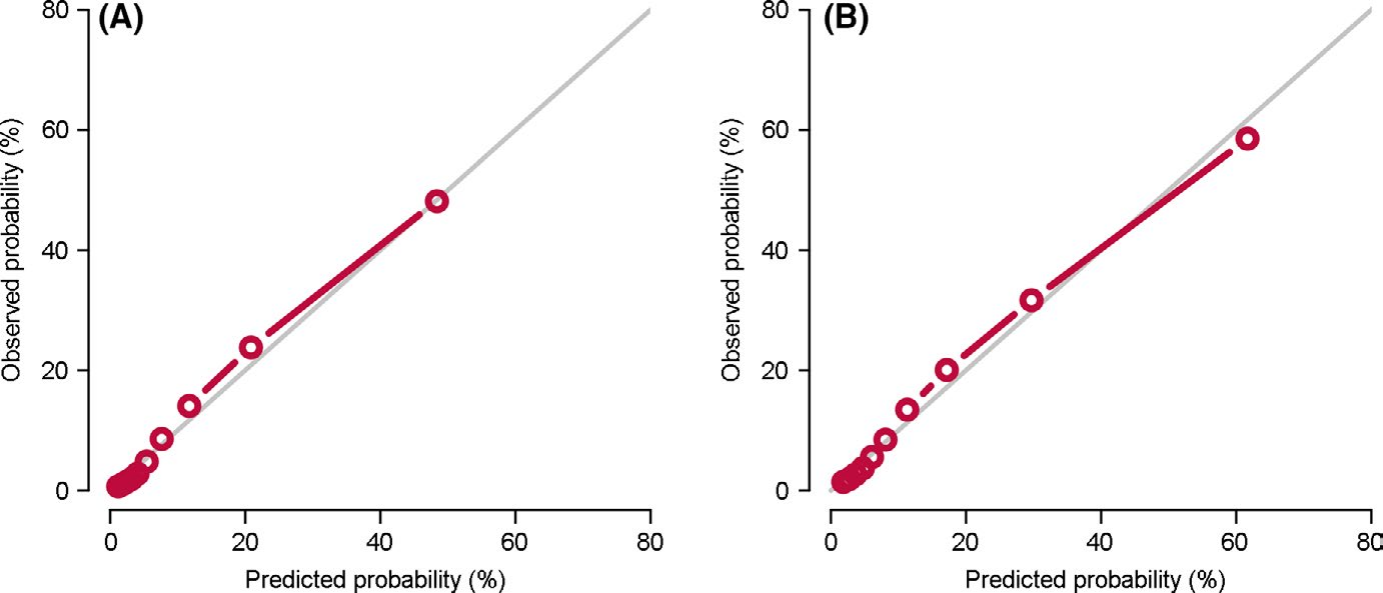
Calibration plots for the competing risk nomogram. The calibration plots for the competing risk nomogram about (A) 3‐ and (B) 5‐year incidence of endometrial cancer specific mortality for endometrial cancer patients treated with hysterectomy

**FIGURE 5:**
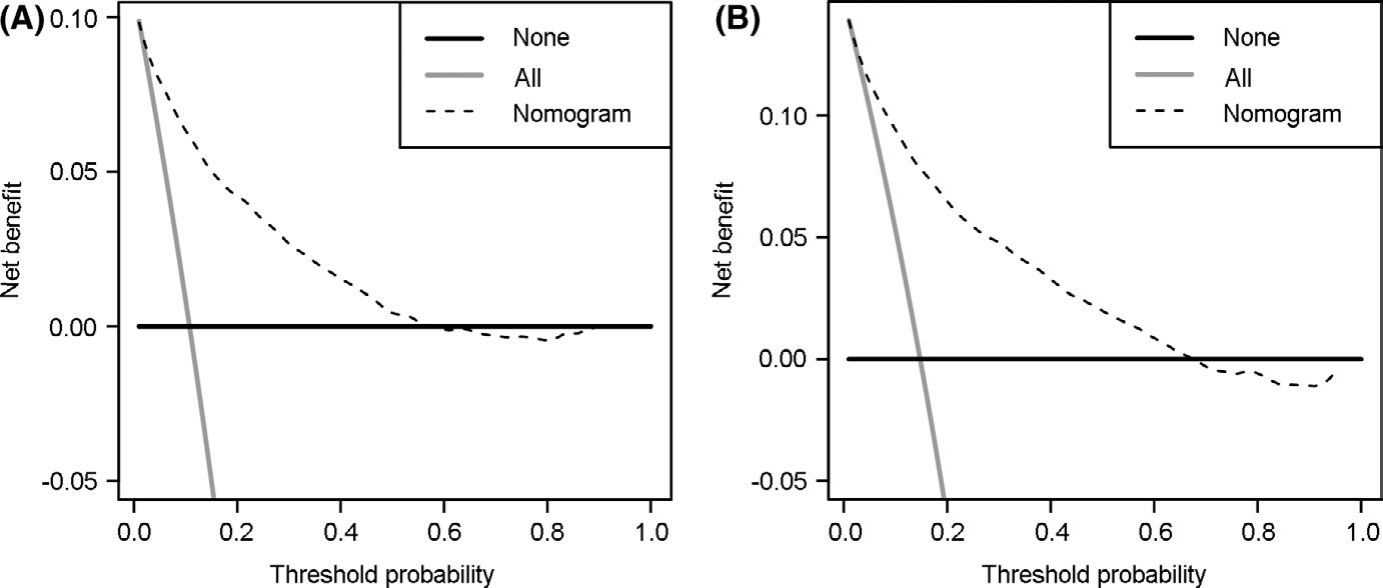
Decision curves for the competing risk nomogram. The decision curves for the competing risk nomogram about cumulative (A) 3‐ and (B) 5‐year incidence of endometrial cancer specific mortality for endometrial cancer patients treated with hysterectomy

The C‐index was 0.857 (95% CI: 0.854–0.859), indicating that the competing risk nomogram could excellently distinguish endometrial cancer‐specific mortality from the censored and other cause‐specific mortality. AUC for the competing risk nomogram in 3‐year prediction of endometrial cancer‐specific mortality were 0.778 (Figure 3A) and that in 5‐year prediction was 0.780 (Figure 3B). The calibration plots for the competing risk nomogram about 3‐year (Figure 4A) and 5‐year (Figure 4B) endometrial cancer‐specific mortality treated with hysterectomy were perfectly fitted and closed to the 45° ideal line. They showed that the predicted mortality from endometrial cancer was consistent with the observed mortality.

### 3.5 Clinical utility

The decision curves of the competing risk nomogram were showed in Figure 5. For predicting cumulative 3‐year incidence of endometrial cancer‐specific mortality (Figure 5A), when the threshold probability was between 1% and 57%, net benefit of the competing risk nomogram was higher than the situations when all patients were treated or none. For predicting cumulative 5‐year incidence of endometrial cancer‐specific mortality (Figure 5B), when the threshold probability was between 2% and 67%, net benefit of the competing risk nomogram was higher than the situations when all patients were treated or none.

## 4 DISCUSSION

We built and internally validated a competing risk nomogram for endometrial cancer patients treated with hysterectomy. Eight prognostic variables, which could be easily obtained from clinical treatment, were finally used to construct model. Its discrimination and calibration were excellent. In addition, the competing risk nomogram had good net benefit.

It was estimated that endometrial cancer accounted for 7% of new cases and 4% of deaths of cancers in female.^17^ There were many researchers conducting related studies about prediction models for endometrial cancer. Some clinical prediction models were built to predict the risk of lymph nodes metastasis, recurrence, and adverse events after surgery of endometrial cancer.^18^, ^19^, ^20^ Another one developed risk‐score models to predict overall survival of endometrial cancer with different grades.^21^ However, most of them performed traditional methods of survival analysis such as Kaplan–Meier method and Cox regression model to analyze survival data. The competitive risks caused by other reasons were ignored. As a result, Kaplan–Meier method would overestimate the risk of interest event and Cox regression model might erroneously estimate the hazard ratio.^6^ When the proportion of the competing event was more than 10%, the Kaplan–Meier method and Cox regression model would cause serious biases.^22^ In this study, the proportion of other cause‐specific mortality was 5.18%, which was less than 10%, and the false positive and false negative results were avoided by competing risk analysis.

Competing risk model, a subdistribution semiparametric proportional hazards model, has been gradually applied to analyze survival data. A big‐data study used it to quantify survival differences of nasopharyngeal cancer and found that other causes of death were important competing risk events for nasopharyngeal cancer patients with advanced age and comorbidities.^8^ Another one found that non‐lung‐cancer‐specific mortality could disturb the prediction of lung‐cancer‐specific mortality, and its impacts increased along with age.^23^ Nevertheless, few studies employed it to build prediction models for endometrial cancer‐specific mortality. In this study, cumulative incidence of function was used to estimate the cumulative incidence of death and the competing risk model was adopted to select prognostic variables. We found that competing risk model could predict the prognosis of endometrial cancer patients treated with hysterectomy without bias. Hence, this new research method could be performed to survival researches with multiple competing outcomes.

In this competing risk nomogram, multiple variables were similar to other related studies of endometrial cancer.^24^, ^25^, ^26^ Compared with FIGO stage, the demographic and tumor‐related information was also taken into consideration, which could quantify the impacts of these factors and predict endometrial cancer‐specific mortality more comprehensively. As expected, grade and FIGO stage of endometrial cancer were the two prominent predictors of the competing risk nomogram. A study also proved that higher grade and FIGO stage were related to worse prognosis of endometrial cancer.^27^ It is because that high grade and FIGO stage indicate high malignance of endometrial cancer, which means that it is easy for tumor to metastasize and spread. We also found that age had critical influence on the prognosis of endometrial cancer. The patients with endometrial cancer have relatively long survival time compared to other tumors, so a proportion of patients might die owing to other causes rather than endometrial cancer. As age rising, elder people are faced with increasing risks of cardiovascular diseases and other systems diseases, which are big threats to the survivorship of elder patients with endometrial cancer.^28^ The employment of competing risk model helps to predict the prognosis of endometrial cancer more precisely, because traditional survival analyses are fit for etiologic research and competitive risk analyses are more suitable for predictive study of diseases with long survival time.^6^

Furthermore, decision curve analysis can assess whether a prediction model or diagnostic test can be actually applied in practice by measuring clinical utility. It has been increasingly used to evaluate prediction models in clinical researches because it integrates the preferences of patients and decision makers into analysis.^14^ And some researches employed it to test the clinical benefit of oncology prediction models.^29^, ^30^, ^31^ However, for endometrial cancer, application of decision curve analysis was still poor. Most prediction studies of endometrial cancer only conducted internal validation but failed to assess their clinical utilities.^18^, ^19^, ^20^ Our study indicated that this competing risk nomogram was of great clinical utility.

Nowadays, clinicians manage endometrial cancer patients with competing risks by qualitative judgment for lacking a comprehensive prognostic assessment tool. With the increasing proportion of elder patients with endometrial cancer, more attention should be paid to conduct effective prediction for their prognosis. Compared with traditional prediction models about overall survival, this robust and easily interpreted competing risk nomogram contained comprehensive factors and could predict time‐related endometrial cancer‐specific mortality more precisely and accurately. Therefore, it was helpful to tailor personalized treatment and stratify risks in order to better manage endometrial cancer patients.

Our study had some advantages compared with other studies. First, the large sample size of this study and the data from the population‐based SEER database could ensure the robustness and universality of the prediction model. Second, the inclusion of demographic and tumor‐related information made our nomogram more comprehensive. Third, the cumulative incidence of endometrial cancer‐specific mortality was predicted more precisely and accurately by controlling the censored and competing events with cumulative incidence function and competing risk model. Finally, decision curve analysis was applied to reflect net benefit of the competing risk nomogram in clinical activities.

Meanwhile, there were still some limitations need to be discussed. First, we failed to include the information of endometrium myometrial invasion depth, lymphovascular invasion, and molecular markers. Besides, commodities and access to healthcare also were critical factors influencing the prognosis of endometrial cancer. However, we found that this competing risk nomogram was still well identified and calibrated under the current factors. Second, because patients in this study came from America and most of them were white, the competing risk nomogram might not be generalizable to all populations. Finally, although nomogram for competitive risk has been internally validated, external validation is still needed to be further performed, which could measure the suitability of the competing risk nomogram among general population.

## 5 CONCLUSION

A competing risk nomogram for endometrial cancer patients treated with hysterectomy was successfully built and internally validated. It was an accurately predicted and clinical useful tool, which could play an important role in consulting and health care management of endometrial cancer patients.

## CONFLICT OF INTEREST

All authors declare that they have no competing interests.

## ETHICS STATEMENT

Ethical approval was not needed in this retrospective study, because all anonymous data was abstracted from the public‐used Surveillance, Epidemiology, and End Results (SEER) database.

## Supporting information

Fig S1

## Data Availability

The data of this study are available from the Surveillance, Epidemiology, and End Results (SEER) database (https://seer.cancer.gov/).
