# Supplementary figures and images for: Competing risk nomogram predicting cancer‐specific mortality for endometrial cancer patients treated with hysterectomy
Source: Cancer Med. 2021 May 1;10(10):3205–13. doi: 10.1002/cam4.3887 (PMC8124128; doi:10.1002/cam4.3887)

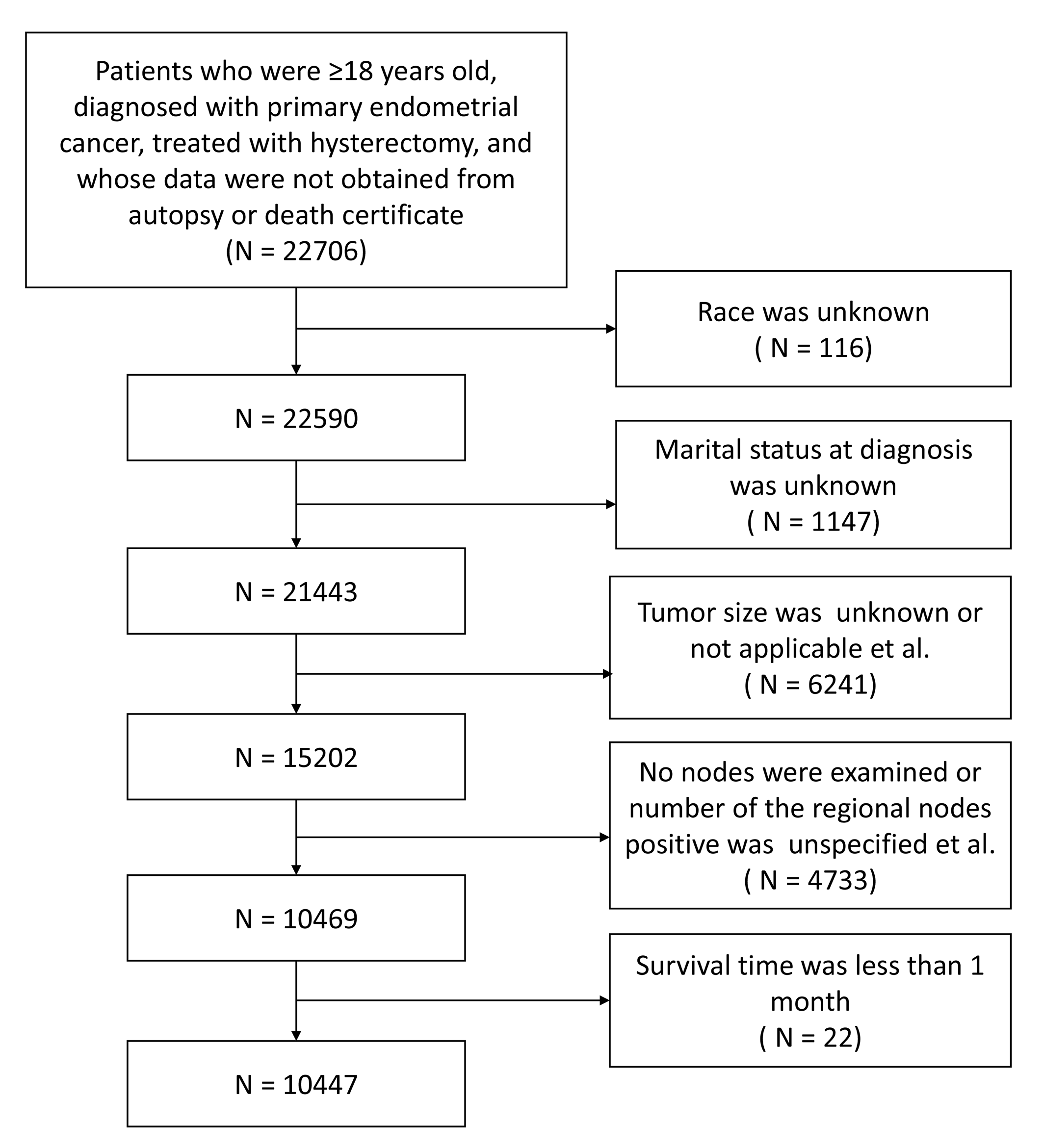

Supplement: Supplementary file 1 — Fig S1 [file CAM4-10-3205-s001.tif]
